# Design of TEMPO-Based Polymer Cathode Materials for pH-Neutral Aqueous Organic Redox Flow Batteries

**DOI:** 10.3390/ma18153624

**Published:** 2025-08-01

**Authors:** Yanwen Ren, Qianqian Zheng, Cuicui He, Jingjing Nie, Binyang Du

**Affiliations:** 1 State Key Laboratory (SKL) of Biobased Transportation Fuel Technology, Department of Polymer Science & Engineering, Zhejiang University, Hangzhou 310058, China; ywren@zju.edu.cn (Y.R.); 22229025@zju.edu.cn (Q.Z.); 22429001@zju.edu.cn (C.H.); 2 Department of Chemistry, Zhejiang University, Hangzhou 310058, China; niejj@zju.edu.cn

**Keywords:** aqueous organic redox flow batteries, polymer cathode material, pH-neutral, TEMPO, high solubility

## Abstract

Aqueous organic redox flow batteries (AORFBs) represent an advancing class of electrochemical energy storage systems showing considerable promise for large-scale grid integration due to their unique aqueous organic chemistry. However, the use of small-molecule active materials in AORFBs is significantly limited by the issue of stability and crossover. To address these challenges, we designed a high-water-solubility polymer cathode material, P-T-S, which features a polyvinylimidazole backbone functionalized with 2,2,6,6-tetramethylpiperidine-1-oxyl (TEMPO) and sulfonate groups. P-T-S exhibits a solubility of 34 Ah L^−1^ in water and 31 Ah L^−1^ in 1.0 M NaCl aqueous solution (NaCl_aq_). When paired with methyl viologen to assemble a pH-neutral AORFB with a theoretical capacity of 15 Ah L^−1^, the system exhibits a material utilization rate of 92.0%, an average capacity retention rate of 99.74% per cycle (99.74% per hour), and an average Coulombic efficiency of 98.69% over 300 consecutive cycles at 30 mA cm^−2^. This work provides a new design strategy for polymer materials for high-performance AORFBs.

## 1. Introduction

Driven by the global energy transition, the development of efficient and sustainable renewable energy systems has emerged as a critical priority for the international community [1,2,3]. While solar and wind energy offer clean and sustainable alternatives, their inherent intermittency and variability pose significant challenges to grid stability [4,5]. In this context, redox flow batteries (RFBs) stand out due to their unique “power-capacity decoupling” feature, which allows the independent scaling of energy storage capacity and power output. This characteristic makes RFBs especially suitable for large-scale, long-duration energy storage application [6,7,8,9].

Traditional RFBs, represented by vanadium RFBs, have achieved initial commercialization [10,11]. However, their extensive application is constrained by challenges, including the scarcity and high cost of metal resources, as well as safety concerns during operation [12,13]. In recent years, the emergence of aqueous organic redox flow batteries (AORFBs) has introduced new possibilities for addressing these limitations [14,15,16]. Among the organic active molecules, 2,2,6,6-tetramethylpiperidine-1-oxyl (TEMPO) has attracted attention as a promising candidate for AORFBs due to its stable redox property [17,18,19,20,21], tunable potential window, and excellent electrochemical reversibility [22,23]. Since the first successful application of water-soluble 4-OH-TEMPO in AORFBs in 2014 [24], researchers have employed a series of molecular design strategies, such as introducing sulfonate groups [25], imidazole groups [21], or quaternary ammonium groups [26,27,28] at the 4-position of the TEMPO molecule, to significantly enhance its solubility in aqueous solutions and improve its electrochemical performance. However, traditional small-molecule electrolytes continue to face critical technical challenges, including severe molecular diffusion losses, high crossover rates through membranes, and insufficient long-term cycling stability [15]. These inherent limitations not only restrict the energy efficiency of the electrolytes but also compromise the reliability and lifespan of the battery systems.

**Figure 1:**
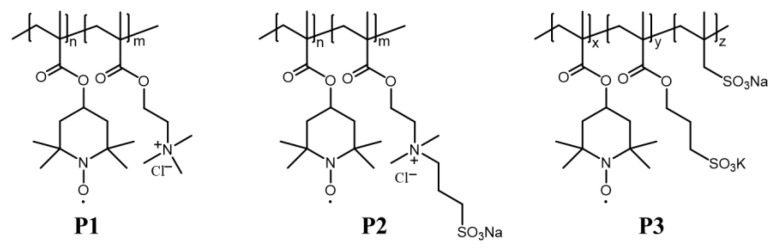
TEMPO-based polymers used in AORFBs [29,30,31].

By leveraging their large molecular size and stable chemical structures, polymer-based redox-active materials can effectively mitigate the undesired problems of crossover and disproportionation. The TEMPO-based polymer for AORFB was first introduced by Schubert’s group [29]. They synthesized polymer P1 (Figure 1), which exhibited a solubility of 10 Ah L^−1^ in 2.0 M NaCl aqueous solution (NaCl_aq_). A P1-based AORFB, with a theoretical capacity of 6.7 Ah L^−1^, demonstrated an average capacity retention rate of 99.87% per cycle over 100 cycles. Subsequently, by introducing sulfonate and ammonium salts, they produced polymer P2 (Figure 1) [30], achieving a solubility exceeding 20 Ah L^−1^ in 1.5 M NaCl_aq_. A P2-based AORFB with a theoretical capacity of 10 Ah L^−1^ showed an average capacity retention rate of 99.71% per cycle over 125 cycles. Fu et al. [31] introduced an anionic terpolymer (P3, Figure 1) through ternary copolymerization, achieving a water solubility of 28.8 Ah L^−1^. A P3-based AORFB, with a theoretical capacity of 12 Ah L^−1^, exhibited an average capacity retention rate of 99.88% per cycle over 170 cycles [31].

In this work, we present a polymer, P-T-S, synthesized via post-polymerization grafting, innovatively utilizing a vinyl imidazole backbone and sulfonate groups grafting to achieve higher solubility (31 Ah L^−1^ in 1.0 M NaCl_aq_). AORFBs were assembled with P-T-S as the cathode and methyl viologen as the anode, using 1.0 M NaCl_aq_ as the electrolyte. At a concentration of 15 Ah L^−1^, the AORFB demonstrates an average capacity retention rate of 99.74% per cycle (99.74% per hour) and an average Coulombic efficiency of 98.69% during 300 cycles at 30 mA cm^−2^ under air atmosphere.

## 2. Materials and Methods

### 2.1. Materials

4-hydroxy-2,2,6,6-tetramethyl-piperidinooxy (4-OH-TEMPO, 98%), 1-vinylimidazole (VIm, 97%), and vitamin C (99.99%), were commercially obtained from Aladdin Chemical Ltd., Shanghai, China. Chloroacetyl chloride (98%), sodium 2-bromoethanesulphoate (SEBA, 98%), 2,2′-azobis (2-methylpropionitrile) (AIBN, 98%), chloroacetic acid (99%), and 4,4-bipyridine (99%) were purchased from J&K Chemical Ltd., Shanghai, China. Dichloromethane (DCM, 99.5%), *N,N*-dimethylformamide (DMF, 99.9%), sodium chloride (99.5%), pyridine (99.5%), and ethyl acetate (99.5%) were procured from Sinopharm Chemical Reagent Co., Ltd., Shanghai, China.

### 2.2. Synthesis of TEMPO-Cl

**Scheme 1:**
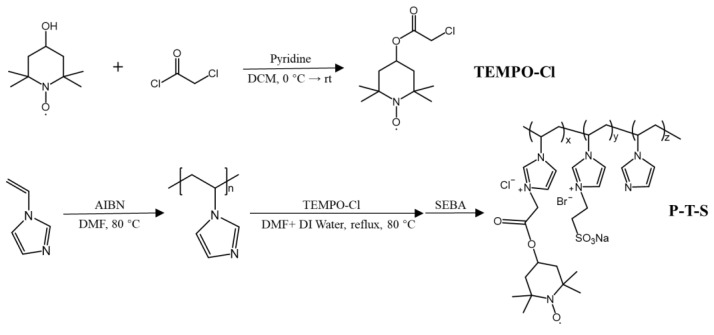
Synthesis routine of target polymer P-T-S.

4-(1-oxyl-2,2,6,6-tetramethylpiperidine)-2-chloroethyl ester (TEMPO-Cl) was synthesized via a nucleophilic substitution reaction (Scheme 1): 4-OH-TEMPO (34.4 g, 0.2 mol) and pyridine (17.4 g, 0.22 mol) were completely dissolved in dichloromethane (DCM, 160 mL) to form a mixed solution. Chloroacetyl chloride (24.9 g, 0.22 mol) was pre-dissolved in DCM (50 mL). After purging with nitrogen for 30 min, the chloroacetyl chloride solution was added dropwise into the mixed solution placed in an ice bath. The mixture was then maintained at an ambient temperature for 24 h to ensure a complete reaction, after which the solid product was isolated via vacuum filtration. After that, the product was washed with DI water, 10% sodium bicarbonate solution, 5% dilute hydrochloric acid, and 10% sodium chloride solution, followed by drying the organic layer with anhydrous Na_2_SO_4_ at an ambient temperature overnight. Finally, the solvent was removed by rotary evaporation, yielding 41.0 g of TEMPO-Cl with a deep red color.

### 2.3. Synthesis of the Polymer P-T-S

The synthesis of the target polymer P-T-S is described as follows (Scheme 1): VIm (18.2 g, 0.2 mol) was completely dissolved in *N,N*-dimethylformamide (DMF, 110 mL), followed by the addition of the initiator azobisisobutyronitrile (AIBN, 0.408 g, 2.5 mmol). After purging with nitrogen for 30 min, the mixture was heated to 80 °C. After 6 h, the product was precipitated and washed three times with ethyl acetate. The resulting white powdery polymer, polyvinylimidazole (PVIm, 17.1 g), was obtained after drying.

Subsequently, PVIm (9.4 g) and TEMPO-Cl (14.9 g, 60 mmol) were sequentially added into a stirred mixture of DMF (200 mL) and deionized water (DI water, 100 mL). The mixture was then heated to 80 °C and allowed to react for 5 days. Following this, SEBA (12.7 g, 60 mmol) was added, and the solution was kept at 80 °C for an additional 3 days. The target polymer P-T-S (20.6 g) was obtained after dialysis and freeze-drying.

### 2.4. Synthesis of MV

The small-molecule anolyte MV was synthesized as follows (Scheme S1) [30]: in 110 mL of DMF, 4,4′-bipyridine (15.60 g, 100 mmol) was dissolved together with chloroacetic acid (25.00 g, 265 mmol). The mixture was then heated to 130 °C and allowed to react for 24 h. After the reaction, the target compound was collected by means of suction filtration and sequentially washed three times with heated DMF, chloroform, and DCM. Finally, the product was dried to yield MV powder (24.60 g) with a grayish-white color.

### 2.5. Structural Characterizationz

The ^1^H-NMR spectra were recorded on a Bruker AVANCE NEO spectrometer (Bruker Corp., Karlsruhe, Germany), and the ^13^C-NMR spectra were recorded on an Agilent DD2-600 spectrometer (Agilent Technologies Inc., Santa Clara, CA, USA), with D_2_O employed as the solvent for both analyses. The molecular weights of the small molecules were measured using an Agilent G6545 QTOF-ESI MS (Agilent Technologies Inc., California, USA), while polymer molecular weights and molecular weight distribution were characterized by a Waters 1525/2414 GPC machine (Waters, Milford, MA, USA) using an aqueous solution of 0.2 M sodium nitrate as the mobile phase. Radical species were captured and qualitatively analyzed using a Bruker A300 EPR Spectrometer (Bruker Corp., Karlsruhe, Germany). FTIR spectra were acquired using a Nicolet 6700 spectrometer (Themo Fisher scientific LLC, Waltham, MA, USA). The solubility of target products was determined by the weight difference method, where 0.5 g of the target polymer was titrated with pure water or sodium chloride solutions of varying concentrations until complete dissolution was achieved, followed by weighing and calculation. Viscosity measurements were performed using a HAAKE MARS 60 rheometer (Thermo Fisher Scientific HAAKE, Karlsruhe, Germany), with temperature-dependent viscosity measurements performed at between 20 and 50 °C with a heating rate of 5 °C min^−1^.

The specific capacity of a polymer (*C*, Ah g^−1^) can be calculated by Equation (1):

(1) $$C=\frac{n\times F}{M}$$

where *n* represents the number of transferred electrons in the polymer, *F* represents the Faraday constant (96,485 C mol^−1^), and *M* represents the molecular weight of the polymer (g mol^−1^).

### 2.6. Electrochemical Characterization

CV tests were conducted using a CHI1660E potentiostat (CH Instruments, Inc., Bee Cave, TX, USA) employing a standard three-electrode configuration comprising a glassy carbon working electrode, a platinum wire counter electrode, and a Ag/AgCl (sat. KCl) reference electrode. For RDE experiments, the CHI1660E potentiostat was also employed, configured with a standard three-electrode setup comprising a glassy carbon working electrode, a Ag/AgCl (sat. KCl) reference electrode, and a carbon rod counter electrode. The scan rate was controlled at 10 mV s^−1^, and the disk electrode rotation speed increased from 300 to 2400 rpm.

### 2.7. Cell Assembly and Measurements

The AORFB system presented in this study primarily consists of a cell stack, two electrolyte storage tanks, and two peristaltic pumps, assembled and operated at ambient temperature in air (Figure S6). The electrolyte storage tanks on both sides are 25 mL conical flasks, connected using PBT tubing. Peristaltic pumps, model LongerPump BT100-1L (Baoding Longer Precision Pump Co., Ltd., Baoding, China), are used in conjunction with a battery testing system, model CT-4008Tn-5V6A-S1-F (Neware, Shenzhen, China). The AORFB was assembled using the following components: a membrane, two graphite felts, two fluororubber electrode frames, two graphite current collectors, two copper current collectors, two PTFE insulating gaskets, and two aluminum end plates. These components were securely sealed together by screws to ensure proper assembly and functionality. The membrane used was a Selemion^TM^ AMVN anion-exchange membrane (AGC Engineering Co., Ltd., Tokyo, Japan) with an effective size of 2.0 cm × 2.0 cm. The graphite felt was pre-treated by calcination at 300 °C for 8 h in a muffle furnace under ambient air atmosphere and cut to dimensions of 2.0 cm × 2.0 cm × 0.3 cm. The XRD results of the graphite before and after calcination indicate that the graphite felt used in this study had a high degree of graphitization before thermal treatment, and the calcination process further improved its graphitization (Figure S7). The operating voltage range was 0.6–1.45 V, with current densities ranging from 10 to 50 mA cm^−2^.

The theoretical capacity of AORFBs (Ah L^−1^) can be calculated by Equation (2):

*Theoretical capacity* = *c* × *specific capacity of polymer* (2)

where *c* is concentration of polymer electrolyte solution (g L^−1^).

## 3. Results

Due to the presence of free radicals on TEMPO, neither the intermediate TEMPO-Cl nor the target polymer P-T-S could be directly characterized by NMR spectroscopy. The chemical structure of TEMPO-Cl was determined by high-resolution mass spectrometry (Figure S1), where the highest peak, observed at m/z 249.1124 in the positive ion mode, matched the theoretical mass-to-charge ratio.

**Figure 2:**
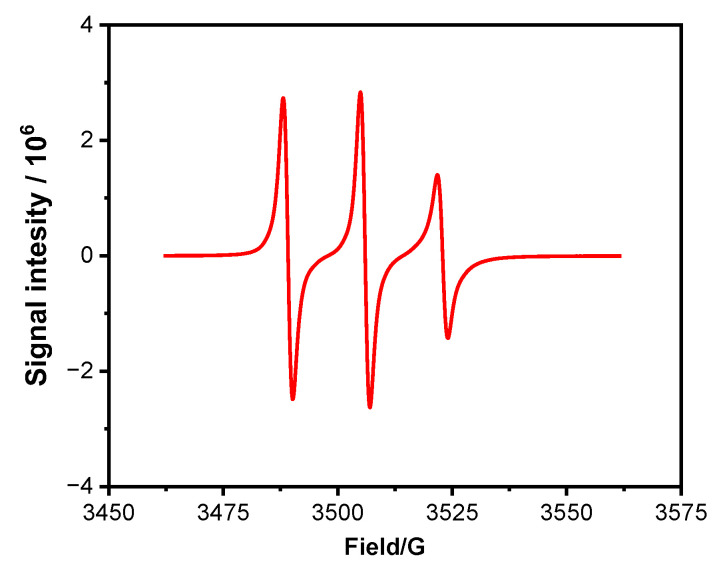
EPR plot of P-T-S.

For the target polymer P-T-S, its number-average molecular weight (*M_n_*) of 12.2 kg mol^−1^, with a molecular weight distribution (*Ð*) of 1.43, was determined by GPC. Subsequent qualitative analysis via EPR (Figure 2) revealed a characteristic triplet signal in the range from 3400 to 3600 G, providing clear evidence that TEMPO radicals were successfully grafted onto the polymer chain. In the triplet signal, the intensity of the third peak is weaker compared to the first two peaks, which may be attributed to interactions between some TEMPO radicals and other functional groups or molecules within the polymer matrix. These interactions alter the electron cloud density of the TEMPO radicals, thereby influencing the intensity of the EPR signal.

**Scheme 2:**
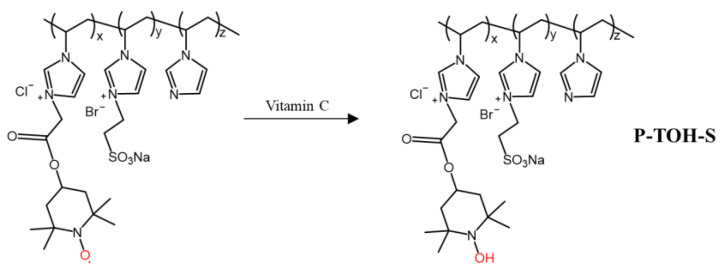
Synthesis routine of the reduced product P-TOH-S.

**Figure 3:**
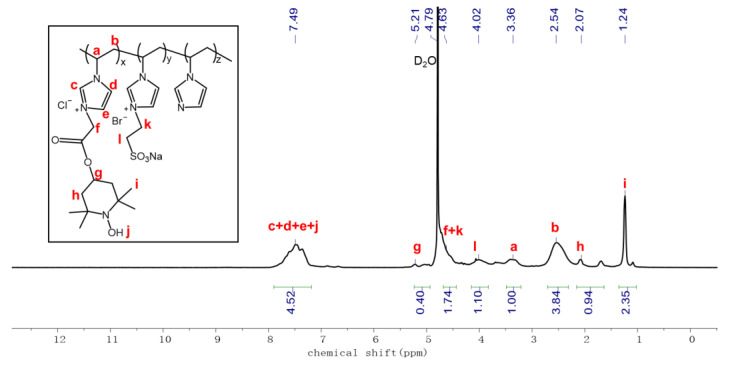
^1^H-NMR spectrum of P-TOH-S in D_2_O.

To quantitatively determine the ratios of x/y/z, the polymer P-T-S was reduced using an excess of vitamin C (ascorbic acid) and then purified to obtain the reduced product P-TOH-S (Scheme 2). The ^1^H NMR spectrum of P-TOH-S (Figure 3) revealed that the ratios of x, y, and z in the polymer are 0.33, 0.5, and 0.17, respectively. The chemical shifts in the observed peaks were assigned as follows: 3.36 ppm (s, br, H_a_), 2.54 ppm (s, br, H_b_), 7.49 ppm (m, br, H_c_, H_d_, H_e_ and H_j_), 4.63 ppm (m, br, H_f_ and H_k_), 5.21 ppm (m, br, H_g_), 2.07 ppm (m, H_h_), 1.24 ppm (s, H_i_), and 4.02 ppm (s, br, H_l_). In the ^13^C NMR spectrum of P-TOH-S (Figure S2), the characteristic peak of the carbonyl carbon (δ = 171.19) further confirmed the successful grafting of TEMPO-Cl. Based on these results, the polymer P-T-S exhibits a theoretical specific capacity of 31 mAh g^−1^. Comparative FTIR analysis was conducted for both the original polymer (P-T-S) and reduced polymer (P-TOH-S), as shown in Figure S3. However, no distinguishable differences were observed between the FTIR spectra of P-TOH-S and P-T-S. Pronounced -OH stretching vibrations (3200–3700 cm^−1^) were exhibited in both spectra. Due to the high hydrophilicity of both polymers and potential water absorption from the atmosphere, the difference between N-O and N-OH groups could not be distinguished by FTIR measurements.

The synthesized MV was also characterized using ^1^H NMR spectroscopy with D_2_O as the solvent (Figure S4). The chemical shifts in the peaks were assigned as follows: 4.49 ppm (s, H_a_), 9.03 ppm (d, H_b_), and 8.50 ppm (s, H_c_). The integration ratios of these characteristic peaks matched the expected ratios of hydrogen atoms at their respective positions, confirming the chemical structure of MV.

**Figure 4:**
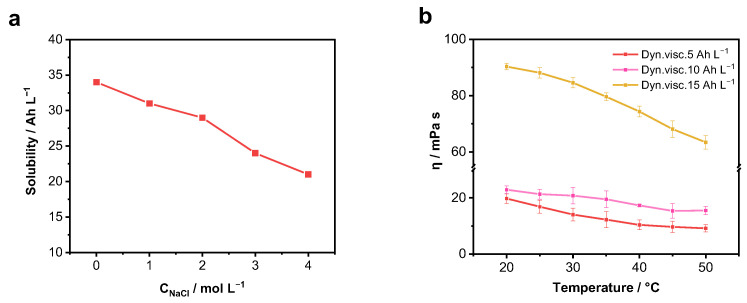
(**a**) Solubility of the polymer P-T-S in NaCl_aq_ of varying concentrations at 20 °C. (**b**) Viscosity of P-T-S electrolyte solutions at different concentrations within the temperature range of 20–50 °C (1.0 M NaCl_aq_ as solvent).

The solubility of the target polymer P-T-S was tested at 20 °C in DI water and NaCl_aq_ of varying concentrations. As shown in Figure 4a, the solubility of the polymer P-T-S decreases as the NaCl concentration increases. In DI water, the solubility of P-T-S reaches 34 Ah L^−1^, while in 1.0 M NaCl_aq_ (simulating the AORFB testing environment), the solubility is 31 Ah L^−1^. In addition, highly concentrated P-T-S in NaCl_aq_ demonstrates low viscosity (for instance, η = 23 mPa s at 20 °C for 10 Ah L^−1^ P-T-S solution in 1.0 M NaCl_aq,_), as shown in Figure 4b and Table S1. The temperature-dependent viscosity measurements reveal a decreasing trend with increasing temperature. These favorable rheological properties, combined with their exceptional solubility, suggest significant potential for enhancing the capacity of P-T-S-based AORFBs.

Furthermore, to investigate the impact of different feed ratios in the quaternization reaction on the target polymer, P-T-S-1 and P-T-S-2 were synthesized (Table 1). However, due to the insufficient number of hydrophilic groups, P-T-S-1 exhibits excessively low solubility in water and is even insoluble in 1.0 M NaCl_aq_. In contrast, P-T-S-2, owing to the low grafting rate of TEMPO, results in a theoretical specific capacity of merely 6.8 mAh g^−1^, which significantly limits its application as an electrode material.

**Figure 5:**
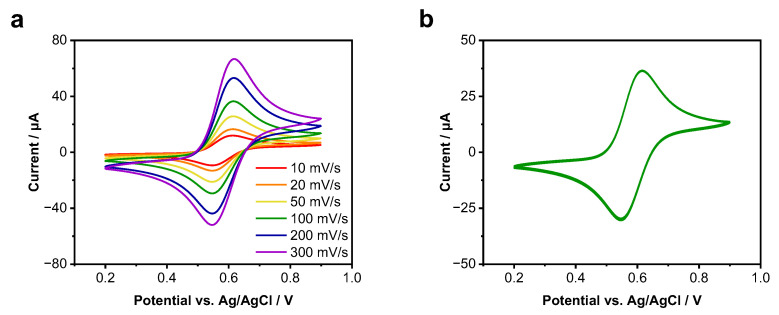
CV curves of the polymer P-T-S (**a**) at a scanning rate range of 10–300 mV s^−1^; (**b**) cyclic stability of 10 consecutive turns at a scanning rate of 100 mV s^−1^.

Subsequently, CV tests were conducted on a 5 g L^−1^ solution of the target polymer P-T-S in 1.0 M NaCl_aq_ at different scan rates. As shown in Figure 5a, CV curves exhibit a typical “duck-bill” shape with good symmetry. At a scan rate of 10 mV s^−1^, we observed an oxidation peak potential of 0.615 V vs. Ag/AgCl, with a corresponding potential difference of 66 mV. With the scan rate increasing, the peak current increases obviously, while the peak potential remains relatively unchanged. This behavior indicates that the electrode reaction exhibits excellent reversibility and a fast reaction rate. Under a constant scan rate of 100 mV s^−1^, the system was subjected to 10 consecutive CV cycles, as shown in Figure 5b. The CV curves overlap almost completely, demonstrating not only the excellent reversibility of the electrochemical reaction but also its high stability over repeated cycles.

**Figure 6:**
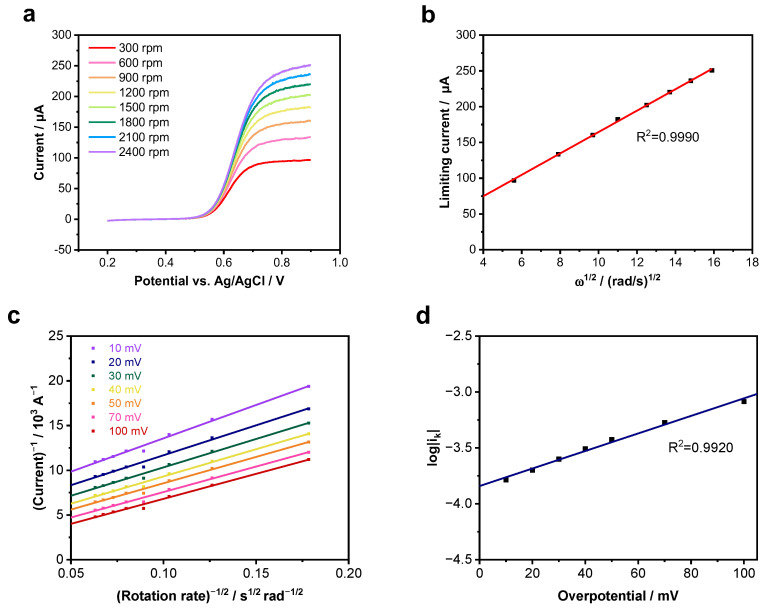
(**a**) Linear sweep voltammograms of P-T-S at a 10 mV s^−1^ scan rate (**b**) Levich analysis depicting the relationship between limiting current and the square root of angular velocity. (**c**) Koutecký–Levich plot showing mass-transfer-independent current (*i_k_*) at various overpotentials. (**d**) Tafel plots.

Linear sweep voltammetry (LSV) was employed to perform RDE tests on the target polymer P-T-S at a concentration of 5 g L^−1^ in 1.0 M NaCl_aq_, as shown in Figure 6a. The Levich plot was constructed by correlating the limiting currents with the square root of angular velocity, demonstrating linear dependence as shown in Figure 6b. Based on the Levich equation, the molecular diffusion (*D*) of redox species was determined to be 2.5 × 10^−9^ cm^2^ s^−1^. At selected rotation speeds, current data were collected at different overpotentials, and the inverse of the current was correlated with the inverse of the square root of the rotation rate (Koutecký–Levich plot, Figure 6c). The intercepts of the fitted curves with the Y-axis provided the kinetic current (*i_k_*) under limiting rotation conditions. Linear regression analysis of the Tafel plot (Figure 6d), derived from kinetic currents measured at different overpotentials, yielded a standard rate constant (*k^0^*) of 3.8 × 10^−5^ cm s^−1^ through Butler–Volmer equation fitting.

The reduction potential of MV was determined to be −0.695 V vs. Ag/AgCl through CV tests, exhibiting a potential difference of 52 mV (Figure S5). Consequently, a theoretical cell voltage of approximately 1.251 V was calculated for an assembled AORFB utilizing the target polymer P-T-S (E_1/2_ = 0.582 V) as the cathode and MV (E_1/2_ = −0.669 V) as the anode. Accordingly, we established 0.6–1.45 V as the operational voltage window for the P-T-S/MV flow battery system to ensure complete redox reactions while maintaining electrochemical stability. A series of P-T-S/MV AORFBs were assembled at air atmosphere, utilizing a 1.0 M NaCl_aq_ electrolyte (Figure S5), with P-T-S acting as the cathode and MV acting as the anode. Considering the principal aim of assessing the viability of P-T-S as an electrode material, the volume of MV solution was set at 1.5 times that of the polymer solution at equivalent concentrations, minimizing the interference of MV side reactions on battery capacity [30].

**Figure 7:**
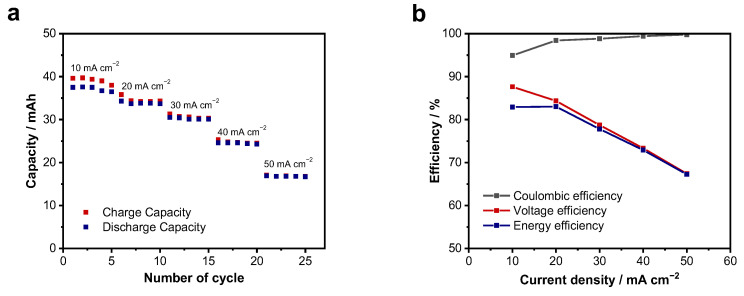
Performance of a 50 mAh P-T-S/MV AORFB at varying current densities with 10 mL of a 5 Ah L^−1^ polymer P-T-S as the catholyte and 15 mL of a 5 Ah L^−1^ MV as the anolyte, corresponding to a theoretical capacity of 50 mAh. (**a**) Charge–discharge capacity; (**b**) Coulombic, voltage, and energy efficiencies.

Initially, an AORFB with a theoretical capacity of 50 mAh was assembled using 1.0 M NaCl_aq_ as the solvent. Specifically, 10 mL of a 5 Ah L^−1^ polymer P-T-S solution and 15 mL of a 5 Ah L^−1^ MV solution were prepared as the catholyte and anolyte, respectively. The battery was subjected to galvanostatic charge–discharge testing within a potential window of 0.6 to 1.45 V and with applied current densities of 10–50 mA cm^−2^ (10 mA cm^−2^ steps) to evaluate its practical capacity and stability across varying current densities. As shown in Figure 7, the battery capacity exhibits a gradual decline with current density increasing. This behavior can be attributed to the electrolyte’s diffusion rate failing to match the reaction rate at higher charge–discharge rates, thereby intensifying concentration polarization within the battery. Taking into account the combined effects of charge–discharge time, capacity retention, and Coulombic efficiency, a current density of 30 mA cm^−2^ was selected as the optimal parameter for the rest battery tests.

Subsequently, a 5 Ah L^−1^ P-T-S/MV AORFB was assembled for further evaluation. Long-term cycling stability was evaluated within a voltage window of 0.6–1.45 V at a current density of 30 mA cm^−2^ (Figure S8). The system delivers an initial discharge capacity of 47.3 mAh, achieving 94.6% utilization of the theoretical capacity. After 200 charge–discharge cycles, the battery capacity decreased to 18.2 mAh, demonstrating an average single-cycle capacity retention rate of 99.69% (99.21% per hour) and an average Coulombic efficiency of 99.06%.

**Figure 8:**
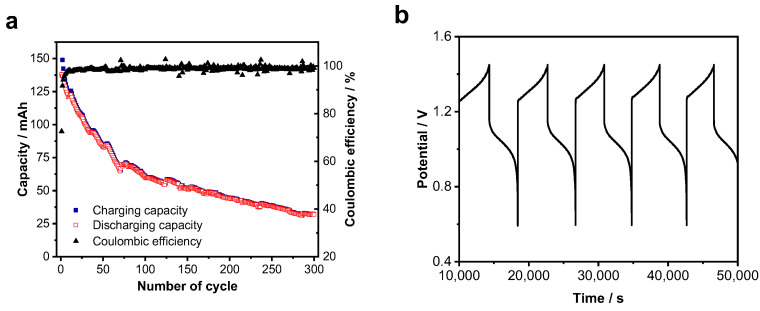
(**a**) Three-hundred charge–discharge cycles of a 150 mAh P-T-S/MV AORFB at a current density of 30 mA cm^−2^; 10 mL of a 15 Ah L^−1^ polymer P-T-S solution and 15 mL of a 15 Ah L^−1^ MV solution were prepared as the cathode and anode electrolytes, respectively, corresponding to a theoretical capacity of 150 mAh. (**b**) Charge–discharge voltage profiles of the battery during the operational period from 10,000 s to 50,000 s.

Following this, the long-term cycling stability of the battery under high-concentration conditions was further investigated. Using 1.0 M NaCl_aq_ as the solvent, 10 mL of a 15 Ah L^−1^ polymer P-T-S solution and 15 mL of a 15 Ah L^−1^ MV solution were prepared as the cathode and anode electrolytes, respectively. An AORFB with a theoretical capacity of 150 mAh was assembled and subjected to long-term cycling tests under identical conditions (voltage range: 0.6–1.45 V; current density: 30 mA cm^−2^). As shown in Figure 8a and Figure S9, the initial discharge capacity of the battery was 138 mAh, corresponding to 92.0% of its theoretical capacity. During the cycling, the Coulombic efficiency started below 90% in the first cycle but rapidly approached 100% from the second cycle onward, which can be attributed to two factors: (1) during initial cycles, trace oxygen and impurities present in the electrolyte are irreversibly consumed through side reactions, while (2) activation processes occur at both electrodes and membrane interfaces. After 300 charge–discharge cycles, the capacity decayed to 32.0 mAh, with 99.74% per cycle capacity retention (99.74% per hour) and an average Coulombic efficiency of 98.69%. Figure 8b displays the charge–discharge voltage profiles during the operational period from 10,000 to 50,000 s. It can be observed that, within the voltage range of 0.6–1.45 V, each cycle exhibits stable and complete charge–discharge plateaus, with no signs of water electrolysis.

**Figure 9:**
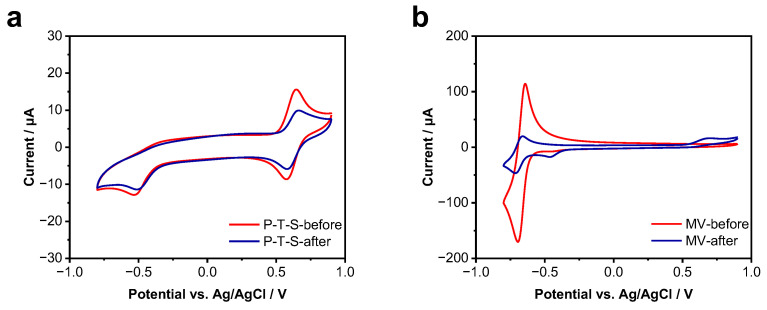
(**a**) CV curves of the pristine and cycled P-T-S electrolyte in AORFB systems. (**b**) CV curves of the pristine and cycled MV electrolyte in AORFB systems.

To investigate the electrochemical stability of redox-active species during battery cycling tests, both the pristine and cycled solutions of P-T-S and MV were diluted to appropriate concentrations and CV tests were performed. The results were compared with the CV curves obtained before cycling. As shown in Figure 9a, the CV curves of the P-T-S electrolyte before and after cycling exhibit minimal changes, indicating no detectable MV crossover. This observation suggests three possible explanations: (1) the anion-exchange membrane partially suppresses cationic MV shuttling, (2) the total cycling duration was insufficient for detectable crossover accumulation, and (3) the MV that shuttled to the other side has undergone side reactions, losing redox activity. The irreversible reduction peak at −0.531 V vs. Ag/AgCl on the anode side is primarily attributed to the presence of the positively charged imidazolium. In contrast, the reversible redox peaks of the MV electrolyte obviously attenuate after cycling, and their shapes also change (Figure 9b), likely due to side reactions such as the second-step irreversible reduction, disproportionation, and hydrolyzation of MV during long-term cycling [32]. Moreover, the gradual pH decrease of the electrolyte during charge–discharge cycling may promote irreversible ring-opening degradation of the TEMPO moiety [33].

## 4. Conclusions

This work presented a novel design strategy for polymer materials in high-performance AORFBs. A novel TEMPO-based polymer P-T-S was synthesized by means of the post-polymerization grafting approach, which exhibited excellent solubility (31 Ah L^−1^ in 1.0 M NaCl_aq_) and favorable rheological properties (23 mPa s viscosity at 10 Ah L^−1^ concentration, at 20 °C). CV tests confirmed its oxidation potential as 0.615 V vs. Ag/AgCl. Additionally, RDE tests revealed a diffusion coefficient (*D*) of 2.5 × 10^−9^ cm^2^ s^−1^ and a reaction rate constant (*k^0^*) of 3.8 × 10^−5^ cm s^−1^. The polymer P-T-S was utilized as the cathode, paired with methyl viologen (MV) as the anode, and 1.0 M NaCl_aq_ was used as the supporting electrolyte to the assemble AORFB. The resulting AORFB had a high theoretical capacity of 15 Ah L^−1^, an average capacity retention rate of 99.74% per cycle (99.74% per hour), and an average Coulombic efficiency of 98.69% throughout 300 consecutive cycles at 30 mA cm^−2^. Notably, the CV curves of the P-T-S electrolyte remained virtually unchanged before and after cycling.

## Figures and Tables

**Table 1:** Impact of different feed ratios on the composition and solubility of the target polymer.

No.	Feed Ratio [VIm]:[TEMPO-Cl]:[SBES]	Unit Ratio in Polymer x:y:z	Solubility in DI Water (Ah L^−1^)	Solubility in 1.0 M NaCl_aq_(Ah L^−1^)
P-T-S	1:0.6:0.6	0.33:0.50:0.17	34	31
P-T-S-1	1:0.9:0.3	0.38:0.21:0.41	0.9	/
P-T-S-2	1:0.3:0.9	0.07:0.76:0.17	7.9	7.6

## Data Availability

The original contributions presented in this study are included in the article/supplementary material. Further inquiries can be directed to the corresponding author.

## Supplementary Materials

The following supporting information can be downloaded at: https://www.mdpi.com/article/10.3390/ma18153624/s1, Scheme S1: Synthesis routine of MV; Table S1: Overview of viscosities of P-T-S with charge-storage capacities of 5, 10, 15, and 31 Ah L^−1^ at temperatures from 20 to 50 °C (in 1.0 M NaCl_aq_); Figure S1: ESI-TOF of TEMPO-Cl; Figure S2: ^13^C NMR spectrum of P-TOH-S in D_2_O; Figure S3. FTIR spectrum of polymer P-T-S and P-TOH-S; Figure S4: ^1^H NMR spectrum of MV in D_2_O; Figure S5. CV curves of MV; Figure S6: The photograph of an assembled AORFB; Figure S7. XRD profiles of graphite felts before and after calcination at 300 °C; Figure S8: (a) 200 charge-discharge cycles diagram of a 50 mAh P-T-S/MV AORFB, at a current density of 30 mA cm^−2^; (b) The corresponding charge-discharge voltage curves during the operational period from 10,000 s to 20,000 s; (c) The corresponding potential-capacity profiles during the 1st, 50th, 100th, 150th and 200th cycles. 10 mL of a 5 Ah L^−1^ polymer P-T-S solution and 15 mL of a 5 Ah L^−1^ MV solution were prepared as the catholyte and anolyte, respectively, corresponding to a theoretical capacity of 50 mAh; Figure S9: Potential-capacity profiles of the 150 mAh P-T-S/MV AORFB during the 1st, 100th, 200th and 300th cycles, at a current density of 30 mA cm^−2^. 10 mL of 15 Ah L^−1^ polymer P-T-S solution and 15 mL of 15 Ah L^−1^ MV solution were prepared as the cathode and anode electrolytes, respectively, corresponding to a theoretical capacity of 150 mAh.

## Abbreviations

The following abbreviations are used in this manuscript:

AORFBs	Aqueous organic redox flow batteries
TEMPO	2,2,6,6-tetramethylpiperidine-1-oxyl
NaCl_aq_	NaCl aqueous solution
RFBs	Redox flow batteries
MV	Methyl viologen
EPR	Electron paramagnetic resonance
CV	Cyclic voltammetry
LSV	Linear sweep voltammograms
RDE	Rotating disk electrode
