# Supplementary material for: Design of TEMPO-Based Polymer Cathode Materials for pH-Neutral Aqueous Organic Redox Flow Batteries
Source: Materials (Basel). 2025 Aug 1;18(15):3624. doi: 10.3390/ma18153624 (PMC12348314; doi:10.3390/ma18153624)
Supplement: Supplementary file 1 [file materials-18-03624-s001.zip › materials-3746827-supplementary.pdf]

Supporting information

Design of TEMPO-Based Polymer Cathode Materials for pH-Neutral Aqueous Organic Redox Flow Batteries

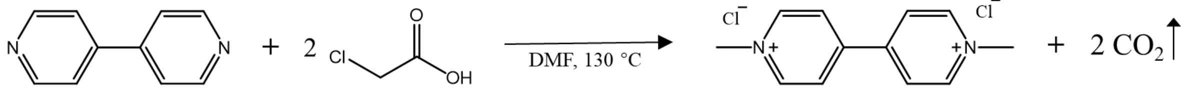

Scheme S1. Synthesis routine of MV.

Table S1. Overview of viscosities of P-T-S with charge-storage capacities of 5, 10, 15 and 31 Ah L^{-1} at temperatures from 20 to 50 °C (in 1.0 M $NaCl_{aq}$[a]).

Temperature [°C]	Dynamic viscosity of 5 Ah L^{-1} [mPa s]	Dynamic viscosity of 10 Ah L^{-1} [mPa s]	Dynamic viscosity of 15Ah L^{-1} [mPa s]	Dynamic viscosity of 31 Ah L^{-1} [mPa s][b]
20	19.8	22.9	90.4	1608.1
25	16.9	21.3	88.1	1086.7
30	14.1	20.8	84.6	969.6
35	12.3	19.5	79.6	785.7
40	10.4	17.3	74.4	631.3
45	9.7	15.4	68.1	492.4
50	9.2	15.5	63.4	385.9

[a] Simulated redox flow battery testing environment. [b] Maximum solubility of P-T-S in 1.0 M $NaCl_{aq}$.

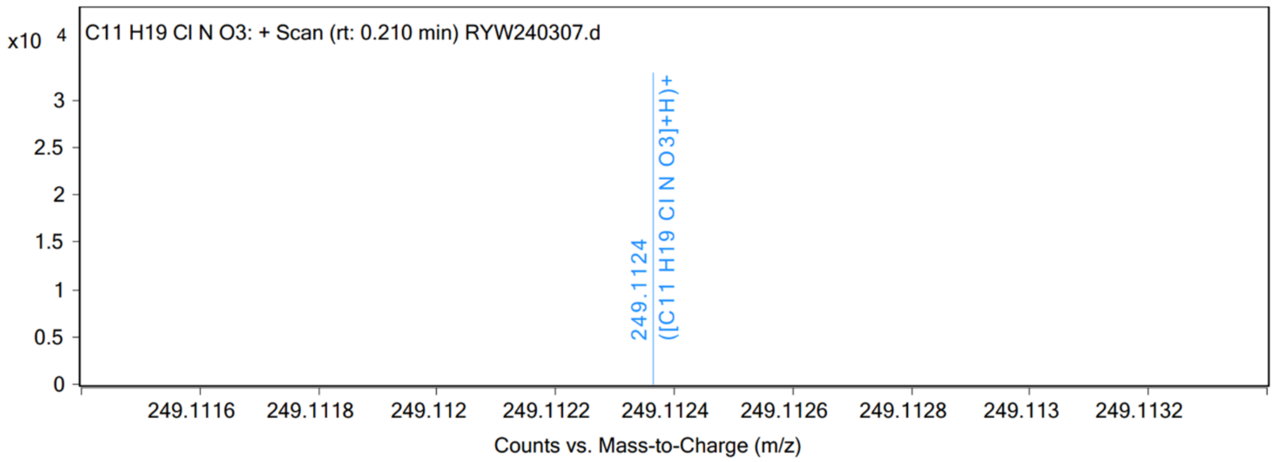

Figure S1. ESI-TOF of TEMPO-Cl.

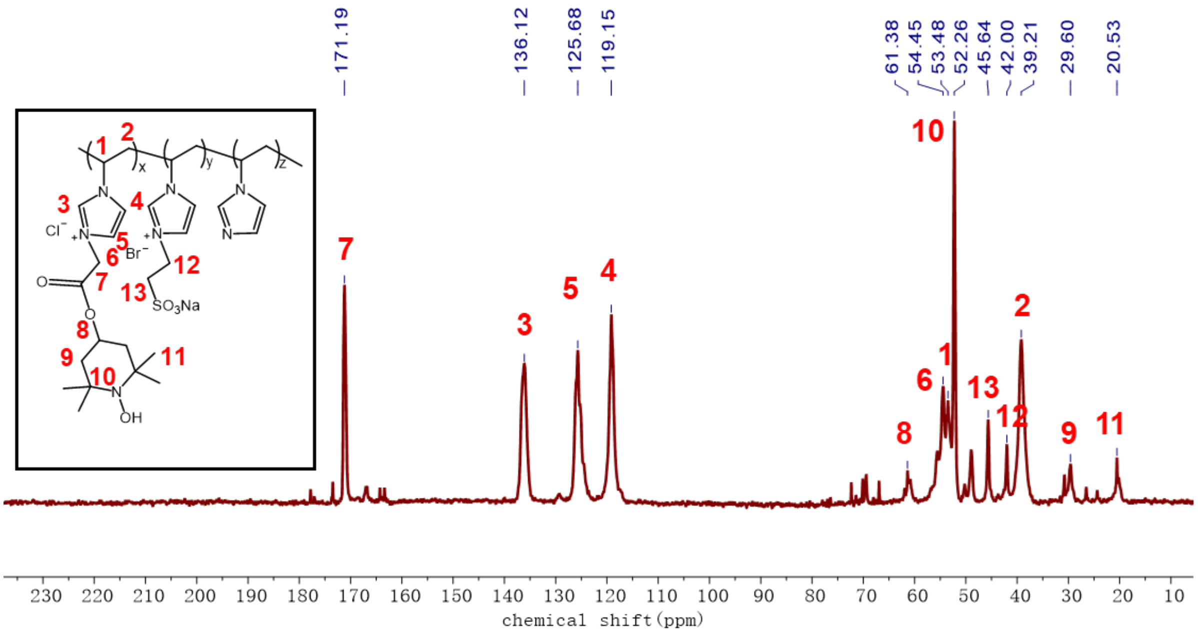

Figure S2. ^{13}C NMR spectrum of P-TOH-S in D_2O.

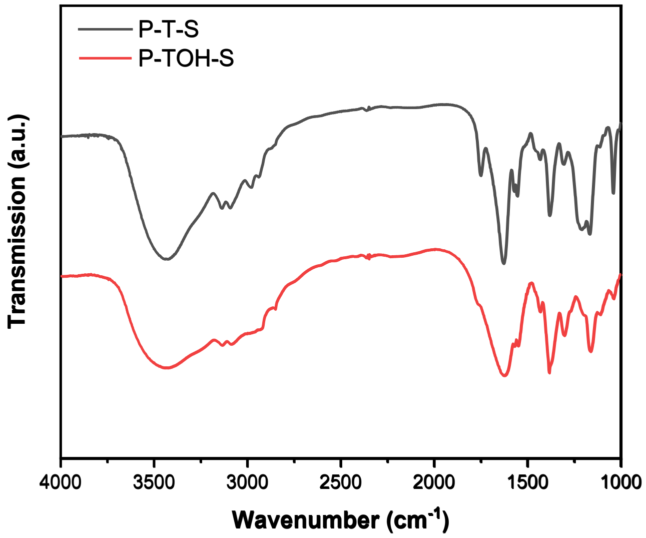

Figure S3. FTIR spectrum of polymer P-T-S and P-TOH-S.

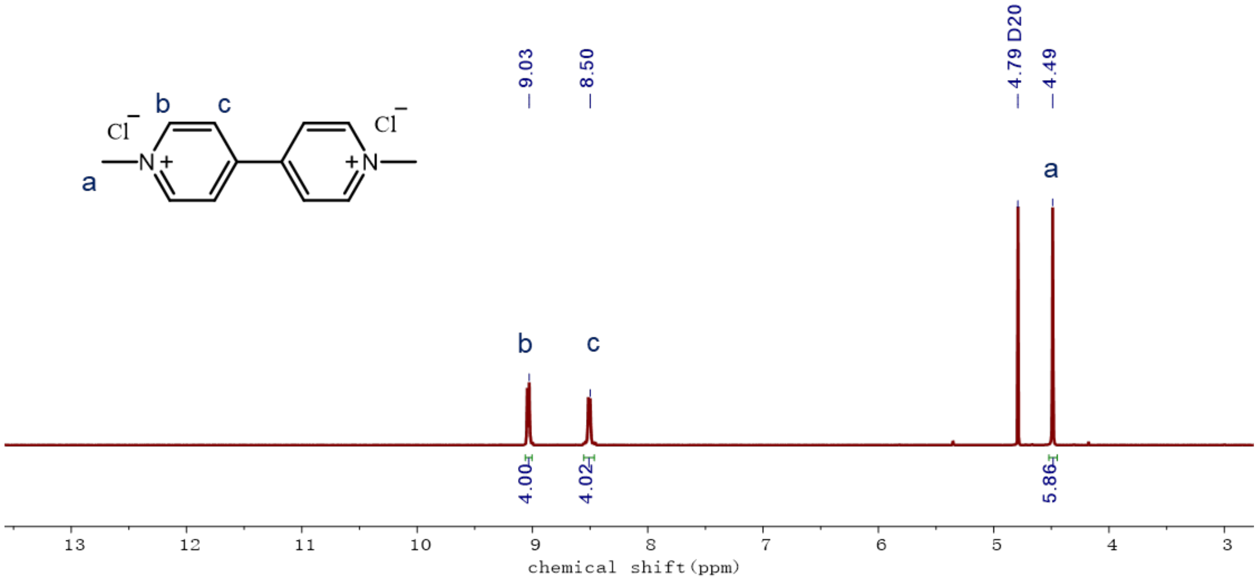

Figure S4. ^{1}H NMR spectrum of MV in D_2O.

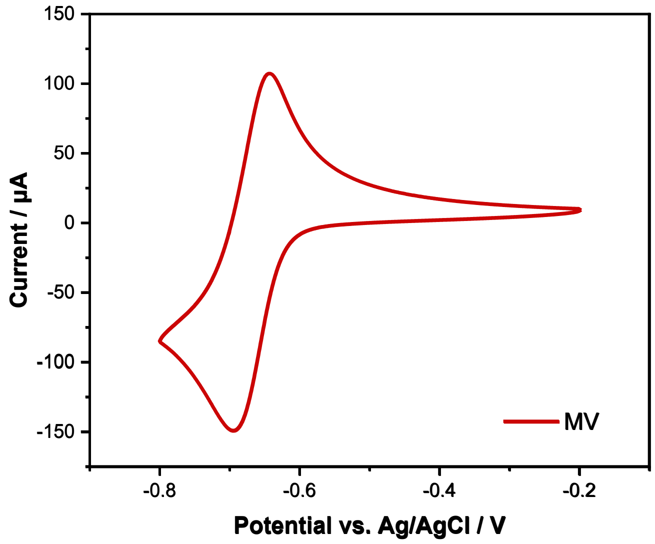

Figure S5. CV curves of MV.

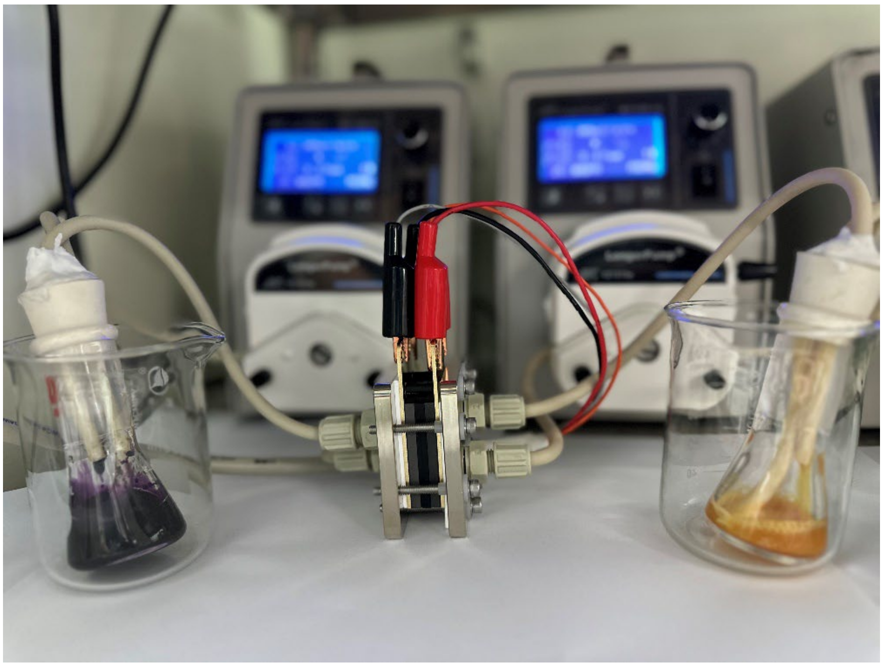

Figure S6. The photograph of an assembled AORFB.

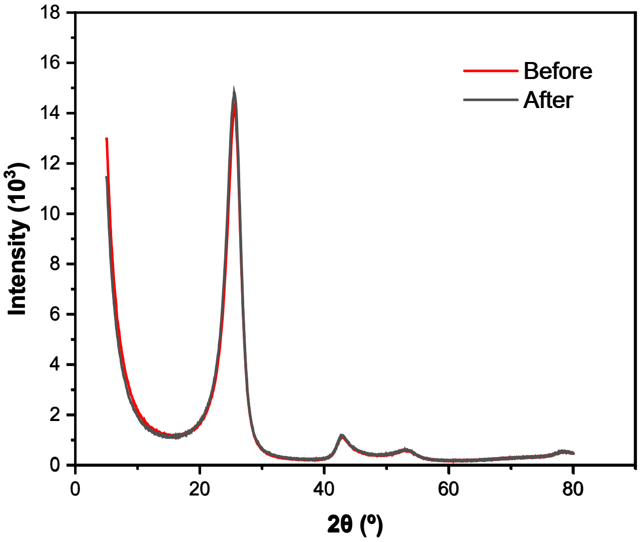

Figure S7. XRD profiles of graphite felts before and after calcination at 300 °C.

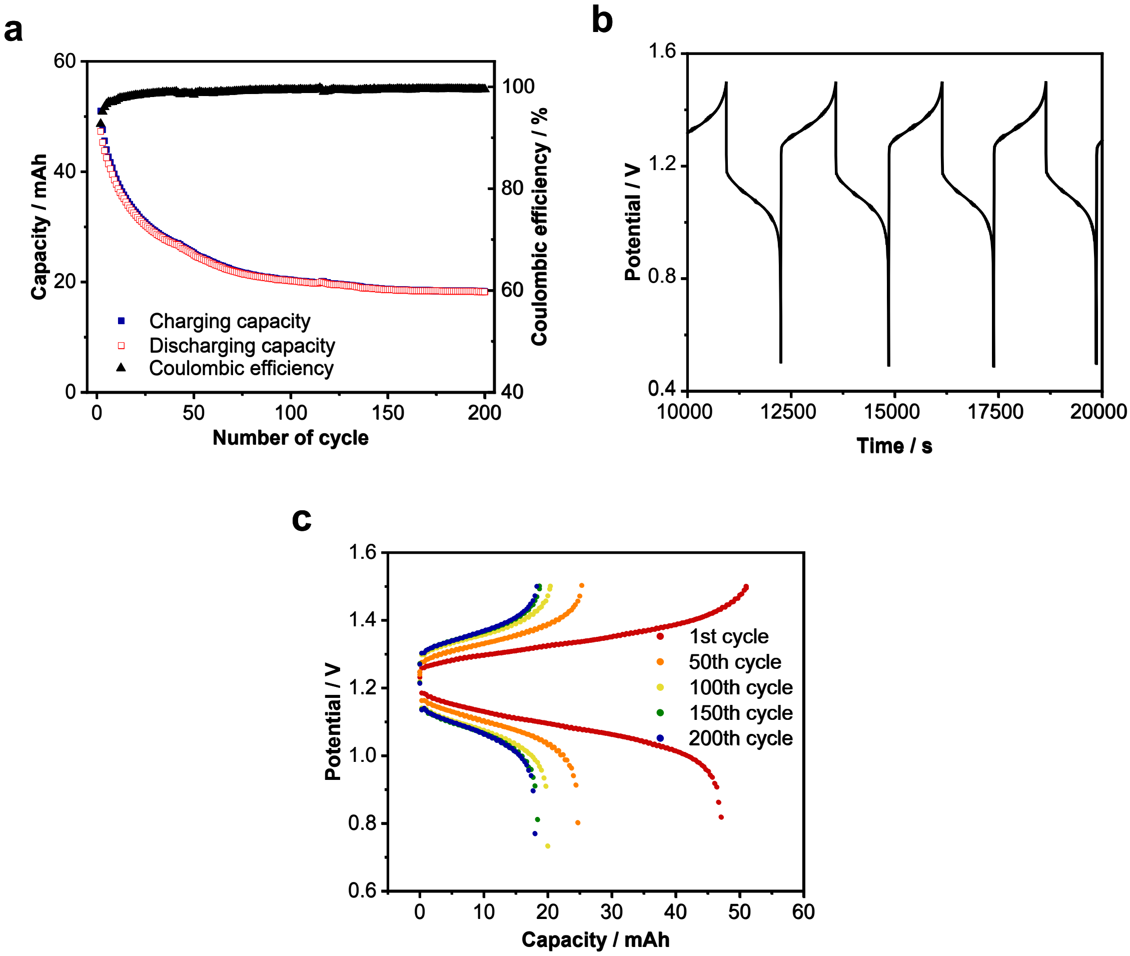

Figure S8. (a) 200 charge-discharge cycles diagram of a 50 mAh P-T-S/MV AORFB, at a current

density of 30 mA cm^{-2}; (b) The corresponding charge-discharge voltage curves during the operational period from 10,000 s to 20,000 s; (c) The corresponding potential-capacity profiles during the 1st, 50th, 100th, 150th and 200th cycles. 10 mL of a 5 Ah L^{-1} polymer P-T-S solution and 15 mL of a 5 Ah L^{-1} MV solution were prepared as the catholyte and anolyte, respectively, corresponding to a theoretical capacity of 50 mAh.

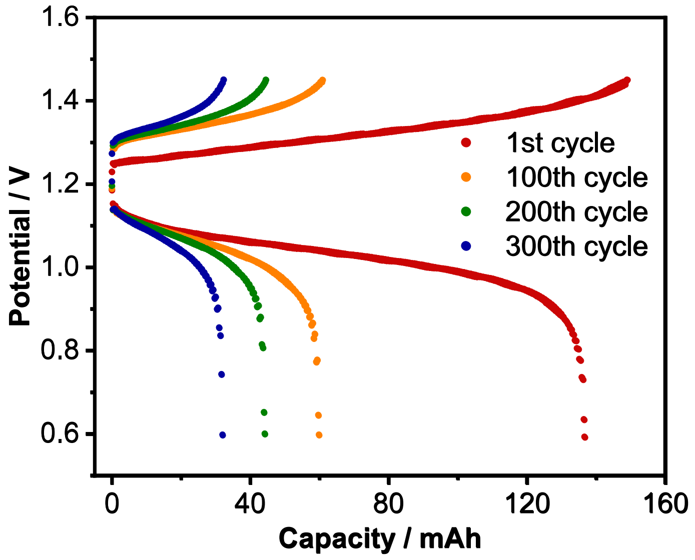

Figure S9. Potential-capacity profiles of the 150 mAh P-T-S/MV AORFB during the 1st, 100th, 200th and 300th cycles, at a current density of 30 mA cm^{-2}. 10 mL of 15 Ah L^{-1} polymer P-T-S solution and 15 mL of 15 Ah L^{-1} MV solution were prepared as the cathode and anode electrolytes, respectively, corresponding to a theoretical capacity of 150 mAh.
